# Recombinant Human Collagen in Clinical Medicine

**DOI:** 10.1111/jocd.71092

**Published:** 2026-08-02

**Authors:** Kyu‐Ho Yi, Jong Keun Song, Hongseok Kim

**Affiliations:** ^1^ Division in Anatomy and Developmental Biology, Department of Oral Biology, Human Identification Research Institute, BK21 FOUR Project Yonsei University College of Dentistry Seoul Republic of Korea; ^2^ Pixelab Plastic Surgery Clinic Seoul Republic of Korea; ^3^ VOS Dermatology Clinic Seoul Republic of Korea

**Keywords:** aesthetic medicine, collagen bioink, photoaging, recombinant humanized collagen, Sihler, type I collagen, type III collagen

## Abstract

**Background:**

Collagen is the dominant structural protein of vertebrate connective tissues and a central biomaterial in skin, cornea, tendon, cartilage, bone, vessel, oral mucosa, and wound repair. Traditional collagen products have largely been extracted from bovine, porcine, fish, or other animal tissues. Although animal‐derived collagens have a long clinical history, they have limitations including batch variability, residual nonhuman epitopes, possible immunogenicity, pathogen‐related concerns, ethical constraints, and limited molecular engineering. Recombinant human collagen has emerged as a synthetic‐biology alternative designed to reproduce full‐length human collagen chains, selected human collagen domains, or engineered humanized collagen‐like sequences in controlled expression systems.

**Objective:**

This systematic review and narrative synthesis summarizes the biological basis, production methods, molecular types, benefits, clinical applications, aesthetic uses, safety considerations, and translational limitations of recombinant human collagen, with emphasis on dermatology, wound healing, corneal regeneration, tissue engineering, injectable aesthetics, and dermal rejuvenation.

**Methods:**

A PRISMA‐informed systematic search was performed across biomedical databases, publisher platforms, and regulatory sources for literature on recombinant human collagen, recombinant humanized collagen, recombinant type I collagen, recombinant type III collagen, recombinant collagen hydrogels, collagen‐based medical devices, dermal fillers, wound healing, corneal substitutes, tissue engineering, photoaging repair, and aesthetic applications. Because the evidence base includes heterogeneous manufacturing papers, in vitro studies, animal experiments, early clinical trials, case series, and regulatory documents, quantitative meta‐analysis was not performed.

**Results:**

Recombinant human collagen offers human‐sequence design, reduced animal‐origin risk, improved batch consistency, scalable manufacturing potential, and engineering flexibility. Type I and type III recombinant collagens are most relevant to dermal, wound, tendon, corneal, and aesthetic applications, whereas type IV, VII, and XVII collagens are increasingly relevant to basement membrane and epidermal adhesion biology. Production remains technically demanding because native collagen requires triple‐helix formation and post‐translational modifications such as proline hydroxylation. Current strategies include yeast expression with prolyl‐4‐hydroxylase co‐expression, microbial production of collagen‐like fragments, mammalian and insect cell systems, transgenic plants, chemical modification, cross‐linked microgels, and collagen‐based bioinks.

**Conclusion:**

Recombinant human collagen is a platform biomaterial rather than a single product class. Its clinical value depends on matching collagen type, molecular format, formulation, degradation profile, mechanical behavior, and biological indication. In aesthetic medicine, its strongest future role may be regenerative matrix restoration rather than simple volume replacement. Larger randomized trials, long‐term safety surveillance, and standardized quality‐control methods are required before broad equivalence with established animal collagens, hyaluronic acid fillers, or biostimulatory injectables can be assumed.

## 1 Introduction

Collagen is the principal structural protein of the extracellular matrix and one of the most important molecular frameworks for tissue integrity. It forms the tensile backbone of skin, tendon, bone, ligament, cartilage, cornea, blood vessels, and many specialized basement membranes. The collagen superfamily includes at least 28 genetically distinct collagen types, each defined by triple‐helical regions and tissue‐specific supramolecular architecture. Fibrillar collagens such as types I, II, III, V, and XI assemble into fibrils that resist tensile stress, whereas type IV collagen forms basement membrane networks, type VII collagen forms anchoring fibrils, and type XVII collagen participates in epithelial adhesion [1, 2, 3, 4, 5, 6, 7].

The defining structural feature of collagen is the triple helix. Collagen chains contain repeating Gly‐X‐Y sequences, where glycine permits tight helical packing and proline or hydroxyproline frequently occupies the X or Y position. Hydroxyproline stabilizes the triple helix at physiological temperature, and its formation depends on prolyl‐4‐hydroxylase activity. Therefore, collagen is not merely a translated amino acid sequence. It is a highly processed structural protein requiring correct chain selection, post‐translational modification, triple‐helix folding, secretion, proteolytic processing, and, for many collagen types, extracellular assembly [2, 6, 7].

Collagen has been used clinically for decades as a biomaterial in hemostatic sponges, wound dressings, skin substitutes, dermal fillers, ophthalmic materials, drug‐delivery systems, and tissue‐engineered scaffolds. Historically, most clinical collagen was extracted from bovine tendon, porcine dermis, fish skin, or other animal tissues. These products remain important, but they are biologically and technologically imperfect. Animal‐derived collagen may contain nonhuman sequence regions, residual telopeptides, species‐specific epitopes, host contaminants, and batch variation caused by tissue source, donor age, extraction method, and purification. It may also raise cultural, ethical, zoonotic, or prion‐related concerns in specific clinical or regulatory settings [8, 9].

Recombinant human collagen was developed to overcome these limitations. In broad terms, recombinant human collagen refers to human collagen chains, human collagen domains, or humanized collagen‐like proteins produced by recombinant DNA technology. A human collagen gene or engineered sequence is introduced into an expression platform such as yeast, bacteria, mammalian cells, insect cells, or transgenic plants. The host produces the collagen or collagen‐like protein, which is then purified, characterized, and formulated as a solution, gel, film, scaffold, microgel, bioink, dressing, or injectable product [8, 10, 11, 12, 13, 14, 15, 16, 17, 18, 19, 20, 21, 22, 23, 24].

The phrase recombinant human collagen can be misleading because it covers many different materials. A full‐length recombinant type I collagen heterotrimer is not the same as a recombinant humanized type III collagen fragment, a collagen‐like peptide, a gelatin‐like digestate, a methacrylated collagen bioink, or a cross‐linked injectable microgel. These products differ in molecular length, triple‐helix stability, cell‐binding activity, degradation behavior, rheology, immunogenicity, and clinical purpose. Regulatory authors have therefore emphasized classification into recombinant human collagen, recombinant humanized collagen, and recombinant collagen‐like proteins [25].

Aesthetic medicine has recently become a major field of interest for recombinant collagen. Skin aging is characterized by dermal collagen fragmentation, reduced fibroblast mechanical signaling, loss of extracellular matrix density, increased matrix metalloproteinase activity, and altered collagen I/III balance [26, 27, 28, 29, 30]. Because recombinant collagen can be designed as a human‐sequence or humanized matrix material, it may have roles in fine wrinkles, photoaging, dermal thinning, post‐procedure healing, acne scars, and injectable skin‐quality improvement. In 2023, China's National Medical Products Administration approved a recombinant humanized type III collagen injection for facial dermal filling of dynamic wrinkles, illustrating the movement of this technology from biomaterials research into aesthetic practice [31].

This review examines recombinant human collagen from biological, clinical, and aesthetic perspectives. It asks how recombinant collagen is made, what types are most clinically relevant, what benefits are plausible, which applications have evidence, and which limitations must be addressed before broad aesthetic and regenerative adoption.

## 2 Methods

This manuscript was prepared as a systematic‐search review with narrative synthesis. The topic spans molecular biology, production engineering, regulatory science, preclinical regenerative medicine, wound repair, ophthalmology, tissue engineering, dermatology, and aesthetic medicine. Because the literature is heterogeneous and does not yet support pooled quantitative outcomes across indications, a meta‐analysis was not performed.

The review was structured according to PRISMA‐informed principles for transparent reporting [32]. Search concepts included recombinant human collagen, recombinant humanized collagen, recombinant collagen‐like protein, human collagen type I, human collagen type III, Pichia pastoris, prolyl‐4‐hydroxylase, plant‐derived recombinant collagen, recombinant collagen medical device, collagen bioink, recombinant collagen dermal filler, recombinant collagen wound healing, recombinant collagen photoaging, corneal substitute, tissue‐engineered skin, and aesthetic medicine. Sources included PubMed/MEDLINE, PubMed Central, publisher databases, ScienceDirect, SpringerLink, Oxford Academic, MDPI, Wiley, regulatory sources, and reference chaining from key reviews and primary research articles.

Included publications addressed at least one of the following domains: collagen biology relevant to recombinant production; expression platforms and post‐translational modification; recombinant collagen characterization; recombinant collagen in wound healing, corneal regeneration, tendon repair, oral mucosa, tissue engineering, or aesthetic medicine; regulatory or quality‐control principles; or comparison with animal‐derived collagen. Excluded literature consisted of papers focused only on non‐recombinant oral collagen supplements, unrelated collagen diseases, general antiaging claims without collagen‐specific data, and cosmetic marketing materials without scientific or regulatory content.

Data were extracted qualitatively across the following categories: collagen type, sequence format, expression platform, post‐translational modification strategy, purification and formulation, indication, biological mechanism, outcome type, safety findings, regulatory relevance, and translational limitations. The review emphasizes mechanistic plausibility, quality of evidence, and clinical decision‐making rather than pooled efficacy values.

## 3 Biological Rationale for Recombinant Human Collagen

The biological rationale for recombinant human collagen begins with the function of collagen as both a structural and signaling molecule. In the dermis, collagen provides tensile strength and determines fibroblast mechanical tension. In wounds, collagen scaffolds support cell migration, granulation tissue formation, angiogenesis, and remodeling. In the cornea, collagen organization governs transparency and mechanical integrity. In tendon, collagen alignment determines load transmission. Therefore, collagen biomaterials are not inert fillers; they influence cell behavior and tissue organization.

The most clinically familiar collagen is type I collagen. It is abundant in skin, tendon, bone, scar tissue, and many connective tissues. Type I collagen provides tensile support and is often used in scaffolds, hydrogels, wound dressings, and tissue‐engineering matrices. Type III collagen is especially relevant to early wound healing, vascular tissues, reticular dermis, and pliable matrix remodeling. The type I/type III collagen relationship is important in skin aging and wound quality because excessive disorganized type I collagen can produce stiffness, whereas type III‐rich early repair may support more flexible matrix formation [1, 2, 3, 4, 5, 26, 27, 28, 29, 30].

Basement membrane and epithelial adhesion collagens are also important. Type IV collagen forms basement membrane networks. Type VII collagen forms anchoring fibrils that link epidermis and dermis. Type XVII collagen is a transmembrane collagen associated with hemidesmosomes and epidermal maintenance. Although most aesthetic products currently focus on type I and type III collagen, future recombinant technologies may increasingly target basement membrane repair, re‐epithelialization, and skin‐barrier restoration.

Recombinant collagen is attractive because it can be designed to reproduce human collagen sequences or selected human collagen‐like domains. This may reduce animal‐origin exposure and increase consistency. However, biological activity depends on molecular form. A full‐length triple‐helical collagen may support fibril assembly, whereas a short humanized fragment may mainly provide cell‐binding or signaling motifs. A cross‐linked microgel may provide mechanical persistence but differ from native collagen in degradation and immune response. Therefore, clinicians and researchers must evaluate each recombinant collagen product as a complete biomaterial, not merely as a familiar protein name.

## 4 How Recombinant Human Collagen Is Made

Production begins with sequence selection. The manufacturer chooses a full‐length human collagen chain, multiple chains needed for a heterotrimer, a homotrimeric collagen type such as type III, a partial collagen domain, a humanized collagen fragment, or an engineered collagen‐like repeat. Full‐length type I collagen generally requires coordinated expression of alpha1(I) and alpha2(I) chains, whereas type III collagen forms homotrimers of alpha1(III) chains. Humanized collagen products may use selected repeated domains from human collagen sequences to improve expression, solubility, or manufacturability [9, 10, 11, 12, 13, 14, 15].

The chosen gene is inserted into an expression vector and transferred into a host system. Yeast systems, especially Pichia pastoris, have been central to recombinant collagen research because they are scalable, suitable for industrial fermentation, and capable of secreting recombinant proteins. However, yeast does not naturally reproduce all mammalian collagen modifications at optimal levels. Studies of Pichia‐based human collagen production demonstrated the importance of co‐expressing human prolyl‐4‐hydroxylase to generate hydroxyproline and stabilize the triple helix [10, 11, 12, 13, 14].

Bacterial systems such as *Escherichia coli* are inexpensive and scalable, but they lack endogenous collagen hydroxylation machinery. They are most useful for collagen‐like peptides, engineered fragments, or systems in which hydroxylation is added through engineering. Bacteria can produce high yields, but endotoxin control is critical for medical and injectable applications. Bacterial production of collagen‐like proteins may be acceptable for some applications, but it should not automatically be equated with full‐length native human collagen [15, 16].

Mammalian and insect cell systems can provide more native‐like post‐translational processing, but they are often more expensive and may be less attractive for bulk biomaterials. Transgenic plants represent another important platform. Human collagen sequences have been expressed in tobacco and maize, sometimes with engineered collagen‐modifying enzymes. Plant‐derived recombinant human type I collagen has been studied for wound healing, skin scaffolds, and corneal implants. Plant platforms can reduce animal‐origin risk and potentially enable scalable production, but they require careful evaluation for plant‐specific impurities, glycosylation patterns, and regulatory identity [17, 18, 19, 20, 21, 22, 23].

The central technical challenge is post‐translational modification. Native collagen requires proline hydroxylation, lysine hydroxylation, selected glycosylation, correct chain registration, and triple‐helix folding. Hydroxyproline is especially important for thermal stability. Without sufficient hydroxylation, a collagen‐like protein may not remain stable at body temperature. Therefore, high‐quality production includes enzyme co‐expression, optimized fermentation, purification of correctly folded species, and analytical confirmation of molecular integrity [7, 10, 11, 12, 13, 14].

Purification must remove host‐cell proteins, nucleic acids, endotoxin, medium components, residual enzymes, aggregates, and degradation fragments. Characterization may include sodium dodecyl sulfate‐polyacrylamide gel electrophoresis, mass spectrometry, amino‐acid analysis, hydroxyproline quantification, circular dichroism, differential scanning calorimetry, electron microscopy, rheology, sterility testing, endotoxin testing, and cell‐compatibility assays. For injectable aesthetic products, rheology, particle size, injectability, collagenase degradation, residual cross‐linker, and tissue reaction become especially important.

**FIGURE 1:**
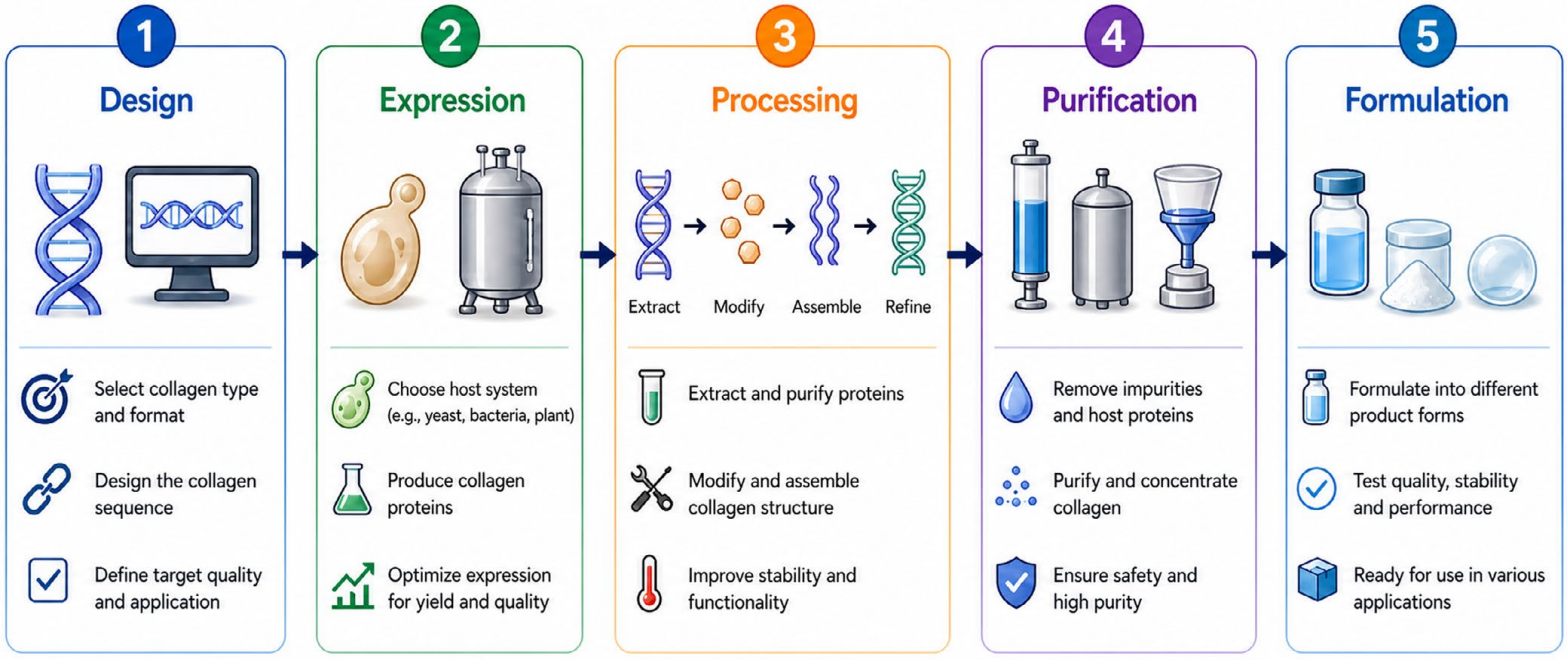
Manufacturing pipeline of recombinant human collagen. Production of recombinant human collagen begins with human collagen or humanized sequence selection, vector construction, expression in an appropriate host system, post‐translational modification, purification, quality control, and indication‐specific formulation. The biological identity and clinical behavior of the final product depend on the entire manufacturing pipeline, not only the collagen sequence.

The purified recombinant collagen is then formulated. It may become a topical solution, lyophilized powder, wound dressing, sponge, flowable gel, cross‐linked microgel, injectable intradermal solution, corneal hydrogel, tendon scaffold, tissue‐engineered construct, or bioink. Every formulation changes clinical behavior. The major stages of recombinant human collagen manufacturing, from sequence selection to indication‐specific clinical formulation, are summarized in Figure 1. A soluble type III collagen injection may degrade quickly and act mainly as a biological matrix signal. A BDDE‐cross‐linked type III collagen microgel may persist longer and behave more like a filler. A methacrylated collagen derivative may be printable but chemically distinct from native collagen.

## 5 Types and Classification of Recombinant Collagen Products

**FIGURE 2:**
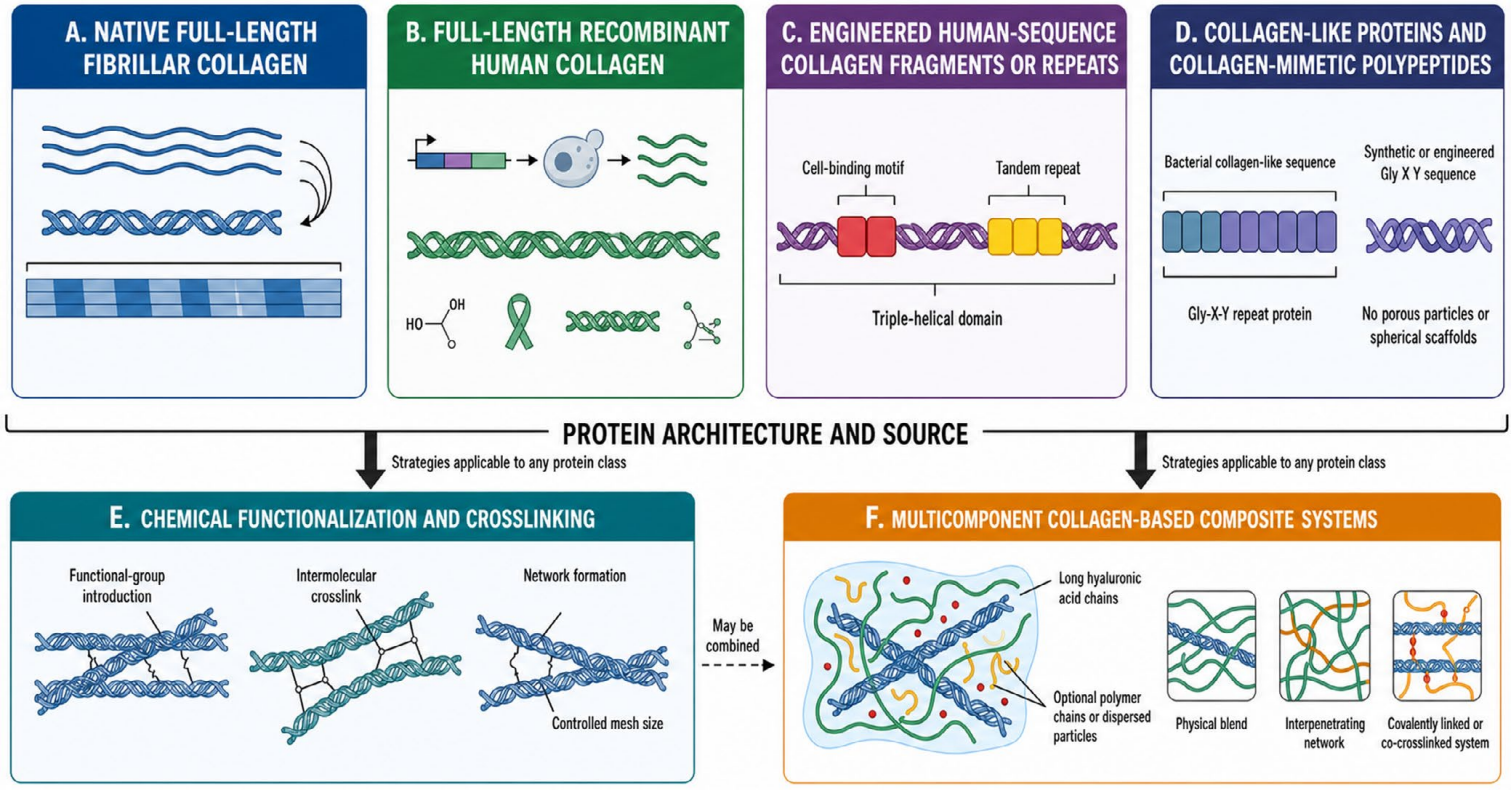
Classification of recombinant collagen products by molecular format and clinical function. Recombinant collagen products include full‐length recombinant human collagen, recombinant humanized collagen, collagen‐like proteins, chemically modified recombinant collagen, and composite systems. These formats differ in manufacturability, biological fidelity, mechanical performance, degradation behavior, and regulatory risk.

A practical classification of recombinant collagen products should combine molecular identity and clinical function (Table 1). The principal recombinant collagen formats and their corresponding clinical applications are illustrated in Figure 2. The first category is full‐length recombinant human collagen. These products attempt to reproduce native human collagen chains and are most biologically faithful. They are important for scaffolds, corneal substitutes, tendon applications, and tissue engineering. The main disadvantages are production difficulty, low yield in some platforms, complex folding, and need for precise post‐translational modification.

**TABLE 1:** Recombinant collagen product classes and clinical implications.

Product class	Typical molecular format	Main advantages	Clinical relevance
Full‐length recombinant human collagen	Native or near‐native human collagen chains, e.g., type I or III	Closest biological similarity to native collagen; scaffold potential	Cornea, tendon, wound scaffolds, tissue engineering
Recombinant humanized collagen	Engineered human sequence‐derived fragments or repeats	Manufacturability, solubility, low animal‐origin risk	Intradermal repair, photoaging, oral mucosa, topical products
Collagen‐like protein or digestate	Short collagen‐like peptides or gelatin‐like materials	High yield, easy formulation, cosmetic flexibility	Cosmeceuticals, coatings, dressings, drug delivery
Chemically modified recombinant collagen	Methacrylated collagen, BDDE‐cross‐linked microgel	Improved mechanics, printability, durability	Bioinks, dermal filler research, long‐lasting scaffolds
Composite recombinant collagen	Collagen combined with HA, GelMA, alginate, cells, PRP, or polymers	Tunable mechanics and biological signaling	Advanced wound care, biofabrication, combination aesthetics

## 6 Benefits of Recombinant Human Collagen

Recombinant human collagen has several theoretical and practical advantages over animal‐derived collagen. The first is human‐sequence design. A recombinant product can be based on human collagen genes or humanized sequences, potentially reducing exposure to species‐specific epitopes. This does not mean immunogenicity is impossible. Immune response depends on folding, purity, aggregates, host‐cell residues, degradation products, cross‐linkers, and route of administration. Nevertheless, human‐sequence design is a major conceptual advantage.

The major recombinant collagen product classes and their corresponding clinical implications are summarized in Table 1.

The second advantage is reduced animal‐origin risk. Animal collagen can be safe when properly sourced and purified, but it carries theoretical concerns regarding viruses, prions, zoonotic contaminants, residual animal proteins, and cultural or ethical limitations. Recombinant collagen avoids direct extraction from animal tissues and can be manufactured in controlled systems. This is attractive for medical devices, tissue engineering, cosmetics, and aesthetic injectables [8, 9, 24, 25, 33].

The third advantage is batch consistency. Recombinant manufacturing allows control over sequence, expression conditions, purification, and release specifications. By contrast, animal‐derived collagen varies by species, age, tissue site, extraction method, cross‐linking state, and purification. Batch consistency is especially important for injectable products and bioinks because small variations in molecular weight, rheology, degradation, or impurities may affect clinical performance.

The fourth advantage is engineering flexibility. Recombinant systems allow the design of specific domains, cell‐binding motifs, protease‐sensitive sequences, charge distribution, solubility, cross‐linking sites, and degradation profiles. This makes recombinant collagen a platform technology rather than merely a replacement material. Products can be tailored for wound repair, injectable dermal remodeling, corneal transparency, tendon alignment, or 3D printing.

The fifth advantage is regenerative potential. Collagen interacts with fibroblasts, keratinocytes, endothelial cells, macrophages, osteoblasts, chondrocytes, and stem cells. In skin, intact collagen supports fibroblast spreading and mechanical signaling. In photoaging, collagen fragmentation reduces fibroblast function and increases matrix degradation. Recombinant collagen may provide a new cell‐supportive matrix, promote collagen I and III expression, improve dermal organization, and support repair after controlled injury [26, 27, 28, 29, 30, 34, 35, 36, 37, 38].

The sixth advantage is compatibility with modern formulation science. Recombinant collagen can be lyophilized, cross‐linked, methacrylated, printed, blended, micronized, or formed into microgels. This allows development of products that are not possible with simple animal collagen extraction. However, engineering must be paired with evidence: a more complex formulation is not necessarily safer or more effective.

## 7 Biological Mechanisms in Skin Repair and Photoaging

**FIGURE 3:**
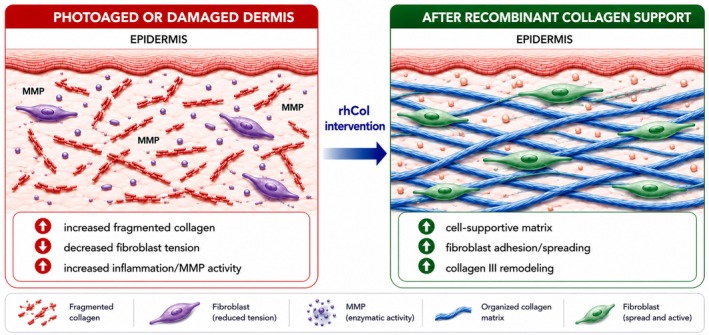
Proposed multiscale mechanisms of recombinant collagen in photoaged and repairing skin. Photoaging is associated with matrix‐metalloproteinase activity, collagen fragmentation, impaired fibroblast spreading, and loss of dermal mechanical organization. Recombinant collagen may provide a provisional cell‐supportive matrix when it reaches viable tissue, but biological effects depend on molecular format, route of administration, persistence, and degradation. Topical application to intact skin should be distinguished from use on a disrupted barrier or by intradermal delivery. Solid arrows indicate established processes; dashed arrows indicate proposed or formulation‐dependent mechanisms that require further clinical validation.

Skin aging provides a useful model for understanding recombinant collagen. Intrinsic aging reduces fibroblast activity, dermal thickness, and collagen turnover. Photoaging accelerates the process through ultraviolet‐induced oxidative stress, inflammation, and matrix metalloproteinase activation. Matrix metalloproteinases fragment collagen fibrils, and fragmented collagen reduces fibroblast attachment and mechanical tension. This creates a self‐amplifying cycle: damaged matrix suppresses fibroblast function, and impaired fibroblasts fail to restore matrix quality [26, 27, 28, 29, 30]. The proposed transition from fragmented, photoaged dermal matrix to a more cell‐supportive remodeling environment after recombinant collagen intervention is illustrated in Figure 3.

Recombinant collagen may interrupt this cycle by providing a provisional matrix. Fibroblasts can adhere to collagen, spread, migrate, and produce new extracellular matrix. In preclinical photoaging models, recombinant humanized collagen type III has been associated with increased collagen I and III expression, extracellular‐matrix remodeling, and improvement in ultraviolet‐induced skin changes [34]. More recent recombinant collagen studies have also reported improved wound healing and repair of photoaging damage [35, 37].

Inflammation is another mechanism. Wound chronicity and photoaging both involve inflammatory signaling. Recombinant collagen may reduce animal‐origin antigen exposure, but it still interacts with macrophages and local immune cells. A well‐designed collagen material may support repair‐associated macrophage behavior, whereas contaminated, overly stiff, or poorly degraded material may provoke chronic inflammation. Early work on recombinant human collagen type III injection suggests possible immunoregulatory effects in photoaging models, but this must be confirmed in larger human trials [37].

Degradation and remodeling are equally important. Collagen should ideally persist long enough to support cell behavior but degrade as new host matrix forms. Products that disappear too quickly may provide only transient hydration or edema. Products that persist too long may cause nodules, stiffness, delayed inflammation, or granulomatous response. Cross‐linked recombinant collagen microgels attempt to solve the durability problem, but their safety depends on cross‐linker chemistry, particle behavior, enzymatic degradation, and tissue response [38].

## 8 Clinical Applications Outside Aesthetic Medicine

Wound healing is among the most biologically coherent applications of recombinant collagen. Chronic wounds often contain degraded extracellular matrix, excessive protease activity, poor fibroblast function, chronic inflammation, infection risk, and impaired angiogenesis. A recombinant human collagen matrix may provide a clean, defined scaffold for cell infiltration, granulation tissue formation, epithelial migration, and remodeling. Plant‐derived recombinant human collagen gels and scaffolds have been evaluated in cutaneous wound repair and diabetic foot contexts, supporting feasibility while highlighting the need for larger controlled trials [22, 23, 39, 40].

Corneal regeneration is another important application. The cornea requires transparent, precisely organized collagenous stroma. Recombinant human collagen type I and type III hydrogels have been fabricated as corneal substitutes with optical properties and epithelial or nerve‐supporting behavior in preclinical studies [41, 42]. A phase 1 clinical study of a biosynthetic corneal substitute demonstrated that recombinant collagen‐based implants could support corneal regeneration, providing one of the most important clinical proofs of concept for recombinant collagen as a regenerative biomaterial [43].

Tendon and musculoskeletal repair also provide a logical target because tendon is collagen‐rich and poorly vascularized. An injectable recombinant human collagen scaffold combined with platelet‐rich plasma has been studied for lateral epicondylar tendinopathy [44]. The concept is attractive because tendon healing requires collagen alignment and matrix remodeling, but load‐bearing applications demand mechanical durability. Recombinant collagen may be best used as a biological adjunct rather than a sole structural replacement unless engineered for adequate tensile strength.

Tissue engineering and 3D bioprinting are rapidly growing areas. Recombinant collagen‐based bioinks can provide a defined cell‐supportive matrix without animal extraction. Recombinant human collagen bioinks have been used for full‐thickness human skin equivalents and engineered tissue constructs [45, 46]. These approaches may enable reproducible in vitro skin models, drug testing platforms, grafts, and eventually vascularized tissue constructs. The main challenges are printability, mechanical stability, degradation, vascularization, and regulatory classification when living cells are incorporated.

**FIGURE 4:**
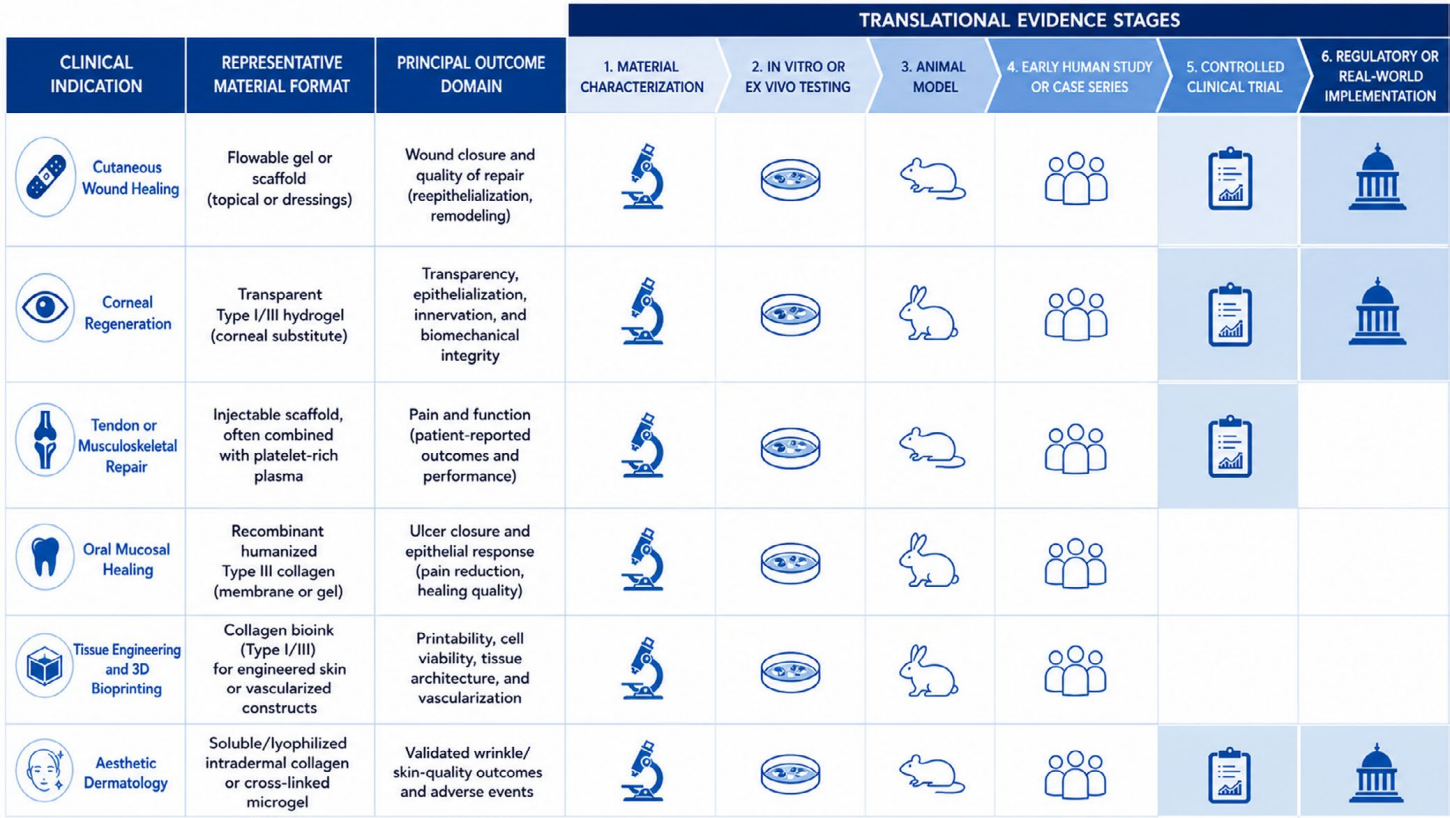
Clinical and aesthetic application map of recombinant human collagen. Recombinant human collagen is a platform biomaterial with potential applications in chronic wound repair, aesthetic dermatology, corneal substitutes, tendon repair, oral mucosal healing, and biofabrication. Each indication requires a distinct molecular format, formulation, degradation profile, and safety evaluation strategy.

Oral mucosal repair is another emerging application. Recombinant humanized type III collagen has been reported to accelerate oral ulcer closure, reduce inflammatory factors, and support human oral keratinocyte behavior [36]. Oral mucosa is mechanically and microbiologically demanding, but rapid epithelialization and inflammation control are clinically meaningful. This indication illustrates how collagen products can be designed for mucosal repair rather than only dermal filler use. The principal clinical and regenerative applications of recombinant human collagen across wound care, corneal repair, musculoskeletal medicine, oral mucosa, biofabrication, and aesthetic dermatology are summarized in Figure 4.

## 9 Aesthetic Applications

Aesthetic medicine is moving from simple filling and paralysis toward biologically oriented skin regeneration. Recombinant collagen fits this trend because it is a matrix material with possible regenerative signaling. However, it should not be marketed as a universal substitute for hyaluronic acid, biostimulators, lasers, or surgery. Its role depends on the problem being treated: fine dermal matrix loss, photoaging, post‐procedure healing, acne scars, dermal thinning, or structural volume loss.

Topical recombinant collagen is attractive for cosmeceuticals and post‐procedure care. Full‐length collagen generally does not penetrate deeply through intact stratum corneum, so claims of deep dermal replacement through intact skin should be cautious. However, topical or mask‐based recombinant collagen may hydrate the surface, improve barrier feel, reduce transepidermal water loss after procedures, and support re‐epithelialization when the barrier has been disrupted by laser, microneedling, radiofrequency, peeling, or abrasion. Cosmetic reviews note growing interest in recombinant collagen because of its biocompatibility, cell‐adhesion properties, and epithelial‐supporting potential [47].

Intradermal recombinant humanized type III collagen is a key aesthetic category. Type III collagen is biologically associated with early wound repair and pliable reticular matrix. Injections may be appropriate for fine rhytids, dermal thinning, photoaging, crepey skin, superficial scars, and skin‐quality improvement. A randomized controlled trial of lyophilized injectable recombinant humanized type III collagen for facial rejuvenation reported clinical evaluation of safety and effectiveness in skin‐aging signs, demonstrating that the field is beginning to move beyond theoretical claims [48].

Injectable recombinant collagen is different from hyaluronic acid filler. Hyaluronic acid provides hydration, viscoelastic volume, and reversibility with hyaluronidase. Recombinant collagen may provide a cell‐supportive matrix and repair signal, but it may be less volumizing unless concentrated or cross‐linked. Therefore, recombinant collagen may be most useful for skin quality rather than major volumetric lifting. Deep folds, skeletal deficiency, and major volume loss may still require hyaluronic acid, calcium hydroxylapatite, poly‐L‐lactic acid, fat grafting, or surgery.

Cross‐linked recombinant collagen microgels are a possible bridge between regenerative collagen and dermal fillers. A 2025 recombinant human collagen type III microgel study described a BDDE‐cross‐linked injectable product with improved thermostability, mechanical strength, and injectability for aging‐skin rejuvenation [38]. This direction is promising, but it also requires rigorous safety evaluation because cross‐linking changes collagen behavior. Residual cross‐linker, gel particle size, rheology, collagenase degradation, nodule risk, inflammatory response, and vascular safety must be evaluated before broad aesthetic use.

Recombinant collagen may also combine well with energy‐based devices. Fractional lasers, radiofrequency microneedling, ultrasound, and microneedling create controlled injury and stimulate remodeling. Applying or injecting recombinant collagen during the repair window may support fibroblast activity and epithelial recovery. However, controlled trials are needed because improvement after combined procedures can be difficult to attribute to the collagen, the device, or their interaction.

Acne scars and atrophic scars are another target. These conditions represent localized dermal matrix deficiency and tethering. Recombinant collagen may act as a scaffold for fibroblast migration and new matrix formation, especially when combined with subcision, microneedling, fractional laser, or radiofrequency. Objective endpoints should include validated scar scales, ultrasound dermal thickness, 3D imaging, optical coherence tomography, and long‐term follow‐up because early swelling can mimic durable improvement.

The aesthetic advantage of recombinant collagen may be most important in patients seeking gradual regenerative change rather than immediate high‐volume correction. It may be particularly relevant in thin skin, photoaged skin, post‐inflammatory dermal damage, fine lines, neck or perioral crepiness, and post‐procedure recovery. The clinical challenge is defining appropriate injection depth, concentration, volume, interval, and combination strategy for each indication.

## 10 Safety, Regulation, and Quality Control

Safety evaluation must address the entire product, not only the word collagen. Important domains include sterility, endotoxin, host‐cell protein residues, nucleic‐acid residues, correct folding, hydroxyproline content, molecular‐weight distribution, aggregates, degradation products, pyrogenicity, cytotoxicity, sensitization, irritation, hemocompatibility, implantation response, chronic inflammation, immunogenicity, and mechanical compatibility [25, 49].

For injectable aesthetic products, additional risks include nodules, granulomas, vascular compromise, infection, delayed inflammatory reactions, migration, overcorrection, undercorrection, and interaction with prior fillers. Products containing cross‐linkers require residual cross‐linker testing, degradation analysis, and long‐term tissue response evaluation. Products produced in bacteria require strict endotoxin control. Products produced in plants require assessment of plant‐derived impurities. Products containing living cells or growth factors may be classified differently from collagen‐only materials.

Regulatory classification depends on composition, intended use, mechanism, and risk. A recombinant collagen dressing, an intradermal injectable, a corneal implant, a bioink, and a cell‐loaded scaffold are not the same regulatory product. Regulatory literature from China specifically classifies recombinant collagen‐based medical devices and distinguishes recombinant human collagen, recombinant humanized collagen, and recombinant collagen‐like proteins [25]. Quality‐control research has further emphasized indication‐specific safety and efficacy verification for collagen‐based medical devices [49].

The NMPA approval of a recombinant humanized type III collagen injection for facial dermal tissue filling is important because it shows that recombinant collagen can be evaluated as a medical aesthetic device rather than remaining only in laboratory research [31]. However, approval in one jurisdiction does not automatically establish approval or equivalence in another. Clinicians must consider local regulatory status, product labeling, training requirements, and adverse‐event reporting systems.

## 11 Comparative Positioning Against Other Aesthetic Materials

Recombinant collagen should be compared with existing aesthetic options in an indication‐specific way. Hyaluronic acid fillers are highly established, mechanically tunable, reversible, and effective for volume, contouring, and hydration. Recombinant collagen is biologically closer to dermal matrix but may not provide the same immediate volume or reversibility. Therefore, it should not be used as a generic hyaluronic acid replacement without evidence for the specific product and indication.

Calcium hydroxylapatite and poly‐L‐lactic acid are biostimulatory materials that induce collagen production through controlled tissue response. They can improve skin thickness and firmness, but their mechanism involves a foreign‐body‐related remodeling response. Recombinant collagen may provide a more physiological matrix cue, although its durability may be shorter unless cross‐linked or formulated as a scaffold. The optimal choice may depend on whether the clinical goal is matrix repair, volume, lifting, thickening, or wrinkle softening.

Autologous fat grafting provides living or partially viable tissue, volume, extracellular matrix, and adipose‐derived regenerative cells, but it is procedure‐dependent and variable. Platelet‐rich plasma and extracellular vesicle products aim to stimulate healing through growth factors or signaling particles but do not provide a collagen matrix by themselves. Recombinant collagen may combine with these therapies, but combination products require careful evidence and regulatory review.

Animal‐derived collagen fillers historically showed that collagen can improve wrinkles, but concerns about allergy testing, immunogenicity, duration, and competition from hyaluronic acid reduced their dominance. Recombinant collagen may revive collagen‐based aesthetics by reducing animal‐origin concerns and improving molecular definition. Yet it must prove durability, safety, and clinical value in modern evidence standards.

## 12 Evidence Synthesis and Current Limitations

The evidence base for recombinant human collagen is promising but uneven. Strong mechanistic evidence supports collagen biology, fibroblast interaction, wound repair, and extracellular‐matrix remodeling. Substantial production literature supports yeast, plant, bacterial, and other expression systems. Translational evidence is meaningful in corneal substitutes, wound healing, and tissue engineering. Aesthetic evidence is expanding rapidly, especially for recombinant humanized type III collagen, but many studies remain early, short‐term, or product‐specific.

A major limitation is heterogeneity. The term recombinant collagen includes full‐length human collagen, humanized collagen fragments, collagen‐like proteins, cross‐linked microgels, bioinks, hydrogels, dressings, and topical products. Evidence for one material cannot be generalized to another. A type III intradermal solution cannot be assumed to behave like a type I corneal hydrogel, and a cross‐linked microgel cannot be assumed to have the same safety profile as soluble recombinant collagen.

Another limitation is the lack of standardized endpoints. Aesthetic trials should include validated wrinkle scales, skin‐quality scales, 3D photography, high‐frequency ultrasound, optical coherence tomography, cutometry, hydration metrics, patient‐reported outcomes, blinded evaluator assessment, and standardized adverse‐event reporting. Wound trials should include complete closure, time to closure, recurrence, infection, pain, quality of healed tissue, and cost‐effectiveness. Tissue‐engineering studies should include mechanical testing, histology, integration, degradation, and functional outcomes.

Long‐term safety is not yet fully defined. Repeated injections, treatment in patients with autoimmune disease, treatment in areas with prior fillers, combination with energy‐based devices, and use in thin or inflamed skin require dedicated study. Cross‐linked formulations require especially careful surveillance because persistence and nodularity risk may differ from soluble products. Post‐marketing registries may be essential for detecting rare delayed inflammatory reactions.

Finally, publication quality varies. Some studies are manufacturer‐supported, small, nonrandomized, or conducted in animal models. This does not invalidate the findings, but it means clinical adoption should be proportional to evidence. The field would benefit from independent multicenter trials and standardized product naming.

## 13 Practical Framework for Clinical and Aesthetic Use

**FIGURE 5:**
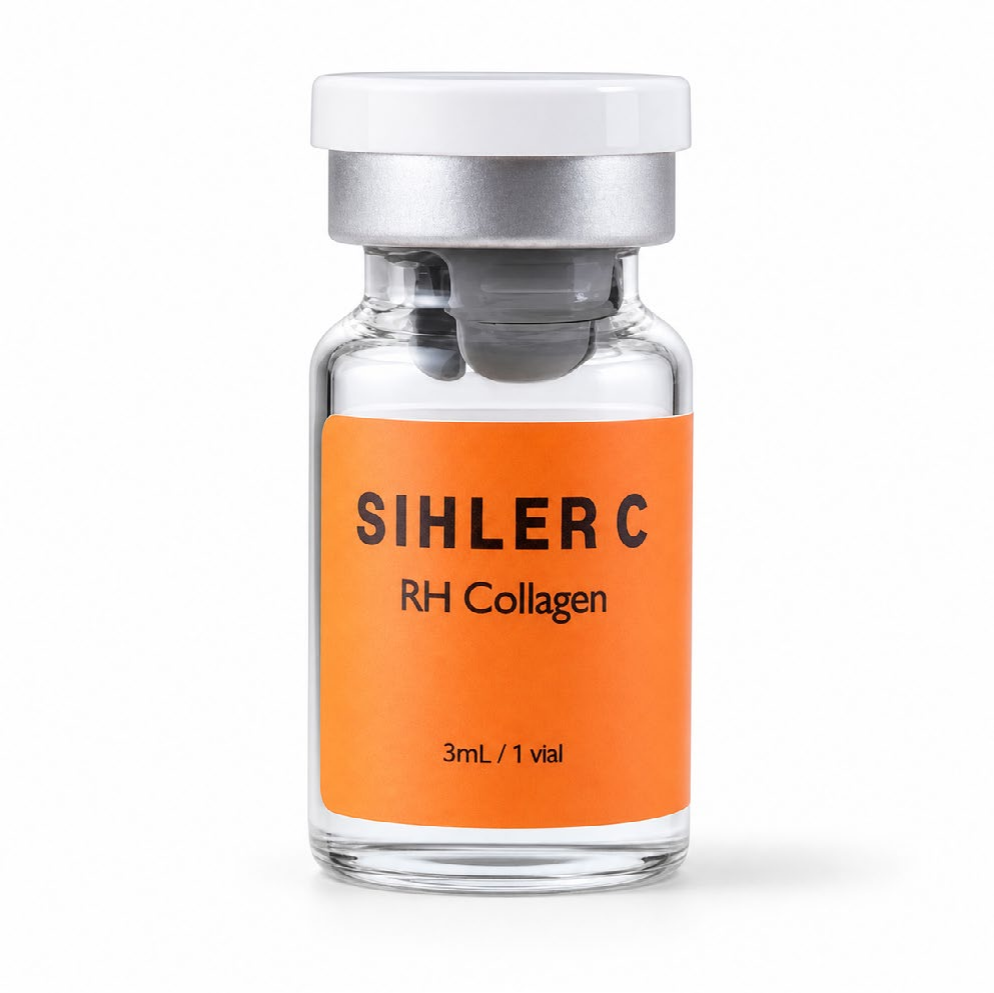
Aesthetic use of recombinant collagen are common nowdays. Aesthetic use should be matched to the clinical problem. Topical or dressing formats may support barrier recovery and post‐procedure care; intradermal recombinant type III collagen may suit fine dermal repair and photoaging; deeper volume loss may require fillers or biostimulators with stronger mechanical profiles. Sihler C (Sihler Inc., Korea) is one of the predominant rh collagen product that consist type III and I.

A practical framework begins with diagnosis of the tissue problem. If the main problem is superficial barrier injury after a procedure, topical or dressing‐based recombinant collagen may be reasonable. If the main problem is fine dermal thinning or photoaging, intradermal recombinant type III collagen may be considered where legally approved and clinically supported. If the problem is deep structural volume loss, recombinant collagen alone may be insufficient unless formulated as a proven filler. If the problem is chronic wound repair, scaffold architecture, exudate management, and protease balance matter more than cosmetic wrinkle improvement. One of the commercial product (Sihler C, Sihler Inc., Korea) that is used now in aesthetic treatments (Figure 5).

The second step is matching collagen type to tissue biology, taking into account the molecular format and clinical purpose of each recombinant collagen product class (Table 1). Type I collagen is logical for tensile scaffolds, tendon, cornea, and structural matrices. Type III collagen is logical for pliable dermal repair, early wound‐like remodeling, and aesthetic skin‐quality improvement. Basement membrane collagens may become important for epidermal adhesion, epithelialization, and barrier repair. Clinicians should not treat all collagen types as interchangeable.

The third step is matching formulation to clinical goal. A soluble collagen solution may provide biological signaling but has a short duration. A lyophilized injectable may be convenient and reproducible. A cross‐linked microgel may improve durability but requires filler‐like safety evaluation. A porous scaffold may support cell infiltration but may not be injectable. A bioink may support engineered tissues but may not be a ready clinical implant.

The fourth step is safety screening. Before aesthetic injection, clinicians should review regulatory approval, product source, indication, injection depth, prior filler history, allergy history, autoimmune disease, inflammatory skin disease, vascular anatomy, and emergency protocols. Recombinant collagen may be safer than animal collagen in some respects, but it is not risk‐free.

The fifth step is objective follow‐up. Because regenerative collagen treatments may produce gradual improvement, photography alone may be insufficient. Ultrasound, cutometry, standardized scales, and patient‐reported outcomes should be used whenever possible. This is especially important for distinguishing durable matrix remodeling from transient edema, hydration, or inflammation.

## 14 Future Directions

The future of recombinant human collagen will likely be precision biomaterials. Instead of one collagen product for all indications, manufacturers may design indication‐specific systems: type III intradermal products for dermal repair, type I scaffolds for wound and tendon support, transparent recombinant collagen hydrogels for cornea, type IV or VII constructs for basement membrane repair, and composite bioinks for engineered skin.

Advanced manufacturing will focus on improving yield, folding, hydroxylation, purity, and cost. Yeast systems may become more efficient through genetic optimization, chaperone engineering, and fermentation control. Bacterial systems may improve through engineered hydroxylation or design of stable collagen‐like sequences. Plant systems may offer scalable animal‐free production. Mammalian and insect systems may remain important where more native post‐translational processing is required.

Product science will focus on mechanical tuning. Aesthetic injectables require defined extrusion force, cohesivity, swelling, degradation, and tissue integration. Wound dressings require absorption, conformability, antimicrobial compatibility, and protease resistance. Bioinks require printability, cell viability, stiffness, and controlled gelation. Corneal implants require transparency, nerve regeneration, epithelialization, and biomechanical stability.

Clinical science must move toward independent, controlled trials. Future aesthetic studies should compare recombinant collagen with saline, hyaluronic acid, microneedling alone, energy‐based treatment alone, and established biostimulators. Wound studies should compare recombinant collagen with standard dressings and animal‐derived collagen. Regulatory science should require consistent nomenclature so clinicians know whether a product is full‐length recombinant human collagen, humanized collagen, collagen‐like protein, or modified composite material.

## 15 Conclusion

Recombinant human collagen is one of the most important emerging biomaterial platforms in regenerative medicine and aesthetic dermatology. It addresses limitations of animal‐derived collagen by enabling human‐sequence design, reduced animal‐origin exposure, improved batch consistency, and molecular engineering. Its biological value is grounded in collagen's central role in extracellular‐matrix structure, fibroblast behavior, wound repair, corneal transparency, tendon function, and dermal aging.

The field is clinically promising but must be interpreted with precision. Recombinant human collagen is not one product. It includes full‐length collagens, humanized fragments, collagen‐like proteins, chemically modified derivatives, cross‐linked microgels, composites, and bioinks. Each has a different safety profile and clinical function. In aesthetic medicine, recombinant collagen should be understood primarily as a regenerative matrix technology, especially for fine dermal repair, photoaging, post‐procedure healing, and skin‐quality improvement. It may evolve into a filler platform, but filler‐like products require rigorous long‐term safety and rheological evaluation.

The next generation of research should combine molecular characterization, objective imaging, histology where ethical, randomized clinical trials, and post‐marketing surveillance. If these standards are met, recombinant human collagen may become a major bridge between biomaterials engineering and biologically oriented aesthetic regeneration.

## Author Contributions

All authors have reviewed and approved the article for submission. Conceptualization: Kyu‐Ho Yi. Writing – original draft preparation: Jong Keun Song and Hongseok Kim. Writing – review and editing: Kyu‐Ho Yi and Hongseok Kim. Visualization: Jong Keun Song and Hongseok Kim. Supervision: Kyu‐Ho Yi.

## Funding

The authors have nothing to report.

## Disclosure

The authors have nothing to report.

## Consent

The authors have nothing to report.

## Conflicts of Interest

The authors declare no conflicts of interest.

## Data Availability

The authors have nothing to report.
